# Outcomes and complications of intraoperative radiotherapy versus external beam radiotherapy for early breast cancer

**DOI:** 10.1002/cnr2.1950

**Published:** 2024-01-11

**Authors:** Vahid Zangouri, Amirhossein Roshanshad, Aliyeh Ranjbar, Mahsa Izadi, Sara Rajaeifar, Ali Goodarzi, Hamid Nasrollahi

**Affiliations:** ^1^ Surgical Oncology Division, General Surgery Department Shiraz University of Medical Sciences Shiraz Iran; ^2^ Breast Diseases Research Center Shiraz University of Medical Sciences Shiraz Iran; ^3^ Student Research Committee Shiraz University of Medical Sciences Shiraz Iran; ^4^ Poostchi Ophthalmology Research Center Shiraz University of Medical Sciences Shiraz Iran; ^5^ Radiation Oncology, Radio‐Oncology Department, School of Medicine Shiraz University of Medical Sciences Shiraz Iran

**Keywords:** complications, cosmetic, EBRT, IORT, radiotherapy

## Abstract

**Background:**

Intraoperative radiotherapy (IORT) is an alternative for external beam radiotherapy (EBRT) for early stage breast cancer (BC). Herein, we compared outcomes, postoperative and post‐radiation complications of IORT and EBRT.

**Methods:**

We conducted a cohort study to compare complications of IORT and EBRT in patients. A checklist of the complications of IORT and EBRT, was used to assess and post‐radiation complications and outcomes.

**Results:**

Overall, 264 women (121 in IORT and 143 in EBRT group) with a mean (SD) age of 55 ± 8.6 years analyzed in this study. The IORT group (quadrantectomy + SLNB + IORT) had more severe post‐operative pain compared to the EBRT group (quadrantectomy + SLNB) (OR = 1.929, 95% CI: 1.116–3.332). Other postoperative complications, including edema, erythema, seroma, hematoma, and wound complications were not significantly different between the IORT and EBRT groups.

EBRT was associated with higher rates post‐radiation complications, including erythema (95.8% vs. 21.5%), skin dryness (30.8% vs. 12.4%), pruritus (26.6% vs. 17.4%), hyperpigmentation (48.3% vs. 9.9%), and telangiectasia (1.4% vs. 0.8%). Multivariate analysis showed that erythema, skin dryness and pruritus, and hyperpigmentation were more severe in the EBRT group, while breast induration was higher in the IORT group (OR = 4.109, 95% CI: 2.242–7.531). Excellent, good, and fair cosmetic outcome was seen in 11.2%, 72%, and 16.8% of the patients in the EBRT group and 29.8%, 63.6%, and 6.6% in the IORT group, respectively, suggesting that the cosmetic outcome was significantly better in the IORT group (*P* < .001). There wasn't statistically significant difference in recurrence‐free survival and overall survival rates between two groups of patients who received either IORT or EBRT (*P* = .953, *P* = .56).

**Conclusion:**

IORT is considered to have lower post‐radiation complications and better cosmetic outcomes in breast cancer patients. Therefore, IORT might be used as the treatment of choice in eligible patients.

## 1 INTRODUCTION

Breast cancer is the most frequent cancer among women, affecting 1.7 million new cases each year.^1^ Currently, breast‐conserving surgery (BCS) is getting popular as it can preserve the breasts, resulting in a better quality of life.^2^ However, the increased likelihood of local recurrence is an important issue for BCS.^3^, ^4^ Postoperative radiotherapy has been proposed to reduce the risk of recurrence and increase the survival in patients who undergo BCS.^5^, ^6^

The standard external beam radiotherapy (EBRT) is continued daily for 5 weeks after BCS. Previous studies have shown that around 21%–30% of patients who had undergone BCS did not complete the recommended radiation therapy.^7^, ^8^ Moreover, EBRT can cause significant acute and chronic complications.^9^, ^10^

Due to the disadvantages of EBRT, intraoperative radiotherapy (IORT) was introduced as an alternative. Administration of radiation at the time of surgery in IORT can help to define the target volume of the tumor cavity more accurately.^3^, ^4^, ^11^ Therefore, IORT enables targeting an area with the highest risk of recurrence and reducing the delivered dose to healthy surrounding tissues and vital organs such as the heart and lungs.^12^, ^13^ Furthermore, shortened overall treatment time, decreased long‐term toxicity, better cosmetic outcomes, lower pigmentation, dryness and swelling of the skin, and reduction in healthcare costs are the other advantages of IORT, which can improve the quality of life of patients.^14^, ^15^ However, IORT is accompanied by some limitations, such as increased surgery time and local recurrence rates.^12^, ^16^

Given the potential advantages of IORT, it is crucial to compare the postoperative and post‐radiation complications associated with IORT and EBRT, as well as their impact on clinical outcomes. There is only limited evidence regarding the rate of local complications of IORT. This study aimed to compare the postoperative and post‐radiation complications of IORT with EBRT groups, and to evaluate their impact on overall survival and recurrence‐free survival in patients undergoing BCS for early‐stage breast cancer. The study assessed complications from the first day until 6 months after surgery and monitored patients for recurrence at least 4 years.

## 2 METHODS

### 2.1 Study design and participants

This study was a retrospective observational study conducted at Faghihi Hospital, which is affiliated with Shiraz University of Medical Sciences (SUMS) between January 2016 and June 2019.

Patients with the following criteria were included: a pathology diagnosis of invasive ductal carcinoma, age older than 45 years, undergoing BCS, a tumor size ≤30 mm in greatest dimension, a negative result of sentinel lymph node biopsy (SLNB) in frozen section, and a positive estrogen receptor (ER) or progesterone receptor (PR) status, as well as either a positive or negative HER2 status (luminal A and B).Exclusion criteria were: tumor size >30 mm in greatest dimension (The maximum tumor size criteria were different based on age, the details of which are mentioned in the study of Zangouri et al.^17^) and a positive SLNB in permanent pathology.

**FIGURE 1:**
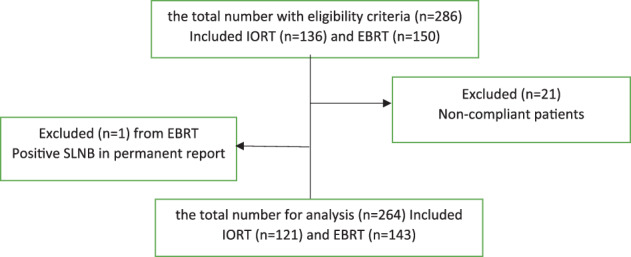
Flow chart of participant inclusion and exclusion.

Patients who met the eligibility criteria for the study, and who were admitted to the surgery ward on the first day after BCS (with IORT (IORT group) or without IORT who required EBRT (EBRT group)), were invited to participate. Those patients who agreed to participate provided their informed consent. Out of the 286 patients who met the eligibility criteria for the study, 267 agreed to participate. Ultimately, 264 patients completed the study. Figure 1 depicts the flow chart of participant inclusion and exclusion.

### 2.2 Surgical procedure

Before the surgery, the hospital obtained consent from all patients to perform the procedure, as well as from patients who chose IORT for the potential use of this method. All patients underwent BCS to remove the lesion and SLNB to evaluate axillary lymph node involvement. Samples were sent to the pathology lab for frozen section pathology evaluation. To prevent infection, all patients in this study were given 500 mg of oral cephalexin every 6 h for 5 days after their surgery.

### 2.3 Radiotherapy

Patients were provided with information about the advantages and disadvantages of both radiotherapy methods before the surgery, and they voluntarily chose the type of radiotherapy they preferred. Patients were aware that their eligibility for IORT would be determined intraoperatively using frozen section pathology reports. If they were not eligible for IORT, the treatment plan would be adjusted accordingly and IORT would not be performed.

**FIGURE 2:**
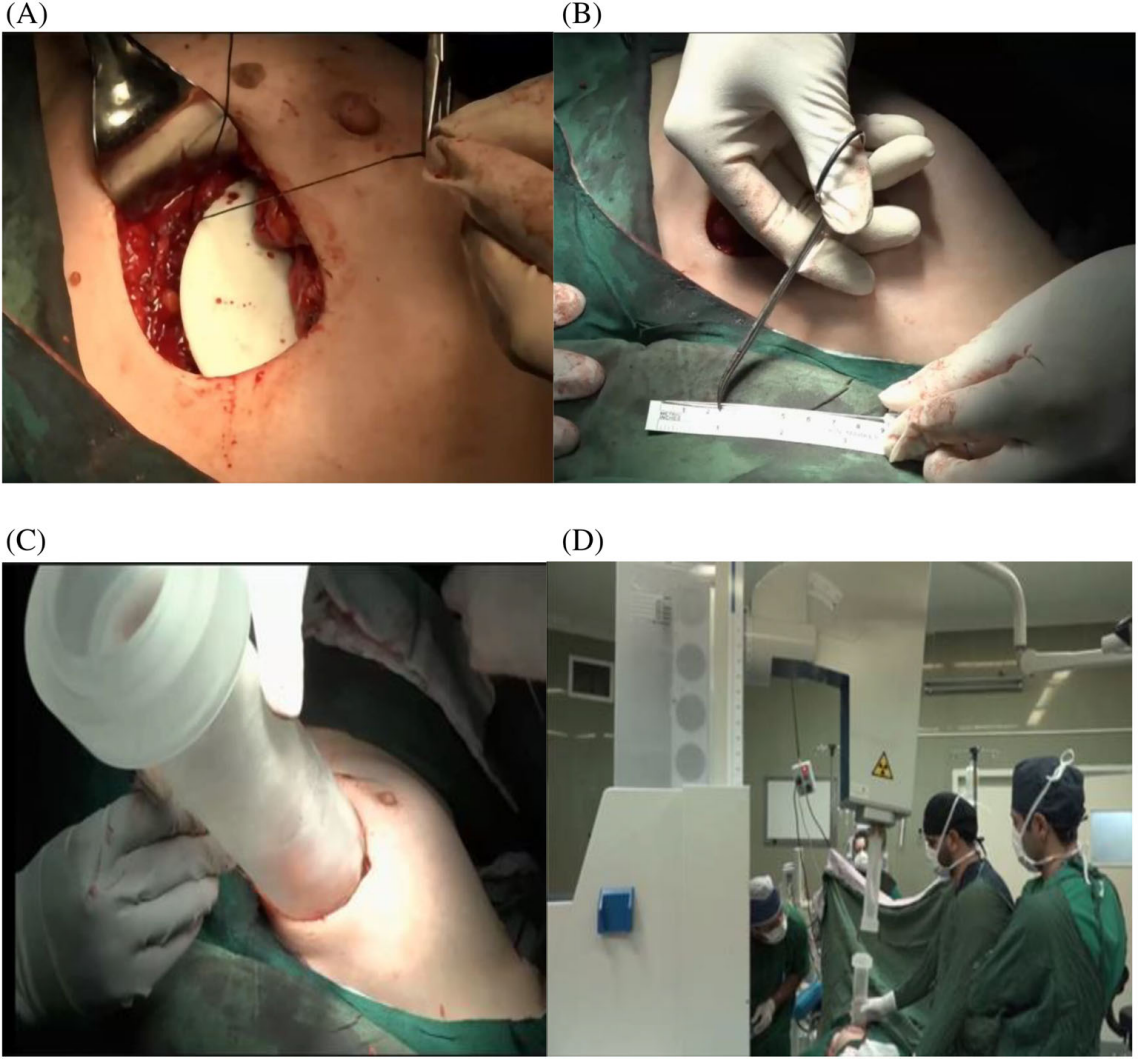
The IORT device being used to treat a patient. (A) Disk insertion, (B) Measuring tissue thickness under radiation, (C) applicator insertion, (D) Connecting the device to the applicator.

At our center, we utilized the Sordina IORT Technologies S.p.A. device from Vicenza, Italia to perform IORT. The IORT procedure involved selecting electrons with energies of 6, 8, 10, or 12 MeV based on the depth of the tumor, which was measured using a marked needle. A dose of 21 Gy was administered to the 95% isodose. The energy of the electrons was chosen based on the thickness of the tissue prepared for radiation therapy. Additionally, the diameter of collimators used in the IORT was selected based on the size of the tumor and the tissue prepared by the surgeon for the procedure. Collimators with diameters of 4–6 cm were used in our IORT procedure. Figure 2 shows an image of the IORT device being used to treat a patient.

In EBRT, the radiation dose was 50 Gy, which was received in 25 fractions. Radiotherapy target volume was whole breast without axillary nodes. Treatment was carried out with 2 tangential fields with 3‐dimensional conformal radiotherapy and our treatment planning system is Prowess.

### 2.4 Data collection and outcomes

Data on demographic features and tumor characteristics were collected from the patients' medical records, stored in the breast cancer research institute. We organized a comprehensive checklist of the complications of IORT and EBRT, adapted from previous studies.^12^, ^14^, ^15^, ^16^, ^18^, ^19^, ^20^, ^21^, ^22^, ^23^, ^24^, ^25^, ^26^, ^27^, ^28^, ^29^, ^30^, ^31^, ^32^, ^33^, ^34^, ^35^ To ensure objectivity, checklists were filled out by trained physicians who were blinded to the type of treatment received by each patient. Each part of the checklist was completed for all patients by a single physician to maintain consistency in the evaluation process. Questions regarding pain and pruritus were filled subjectively by the patients. Items of this checklist are categorized in two parts:

1. Evaluation of acute and sub‐acute complications after surgery: Data of the two groups of the patients, EBRT (quadrantectomy + SLNB and required EBRT) versus IORT (quadrantectomy + SLNB + IORT), were obtained three times after the operation. A total of 24 h after the operation: pain; One week after the operation: pain, edema, erythema, palpable seroma, hematoma, pruritus, skin dryness, and wound complications including wound infection and dehiscence; Three weeks after the operation: pain, healing delay, retraction of scar.
2. Evaluation of acute and subacute complications and outcomes of radiotherapy. We collected data of the patients who received IORT or EBRT at two different times after radiotherapy. One week after the end of the radiotherapy: erythema, skin complications including skin toxicities, breakdown, and necrosis; Six months after the end of radiotherapy: telangiectasia, pigmentations, breast induration, and cosmetic outcomes according to standards set forth by the Harvard criteria as shown in Table 1.

**TABLE 1:** Breast cosmesis scale.

Harvard scale (4‐point Likert scale)	
Excellent	Treated breast nearly identical to untreated breast
Good	Treated breast slightly different from untreated breast
Fair	Treated breast clearly different from untreated breast, but not seriously distorted.
Poor	Treated breast seriously distorted.

### 2.5 Statistical analysis

The study analyzed the outcomes and complications associated with IORT and EBRT in patients with early‐stage breast cancer by SPSS software (SPSS Inc., Chicago, IL), version 21.0. Mean and standard deviation (SD) were used to present continuous variables, while frequency and percentages were used for categorical variables. These descriptive statistics helped to summarize the demographic and clinical characteristics of the study population, including age, tumor size, and radiotherapy method. The chi‐squared test was used to compare the rate of complications between the IORT and EBRT groups. The *t*‐test was used to compare quantitative variables, such as age and tumor size between the IORT and EBRT groups. Binary logistic regression was used to evaluate the association of age, tumor size, diabetes, and type of radiotherapy with postoperative and post‐radiation complications. The backward method was used to perform logistic regression, which helped to identify the most significant predictors of complications and adjust for potential confounders. Odds ratio (OR) with its 95% confidence interval (CI) were used to present this association, which helped to quantify the strength and direction of the relationship between the predictor variables and the outcomes. Kaplan–Meier Plots: Kaplan–Meier plots were used to compare overall survival and recurrence‐free survival between the IORT and EBRT groups.

### 2.6 Ethical considerations

Our study was conducted in accordance with the declaration of Helsinki. Ethics committee of Shiraz University of Medical Sciences approved this study (ethical code: IR.SUMS.MED.REC.1400.078). The patients gave their informed and written consent to participate in this study.

## 3 RESULTS

A total of 264 women with early‐stage breast cancer were evaluated in this study. One hundred twenty‐one of the patients received IORT, and 143 received EBRT. The participants' mean age was 55.7 years (SD 8.1) in the IORT group and 54.3 years (SD 8.9) in the EBRT group. Tumor mean size was 1.53 cm (SD 0.55) and 1.74 cm (SD 0.64) in IORT and EBRT groups, respectively (*P* = .005). (Table 1).

**FIGURE 3:**
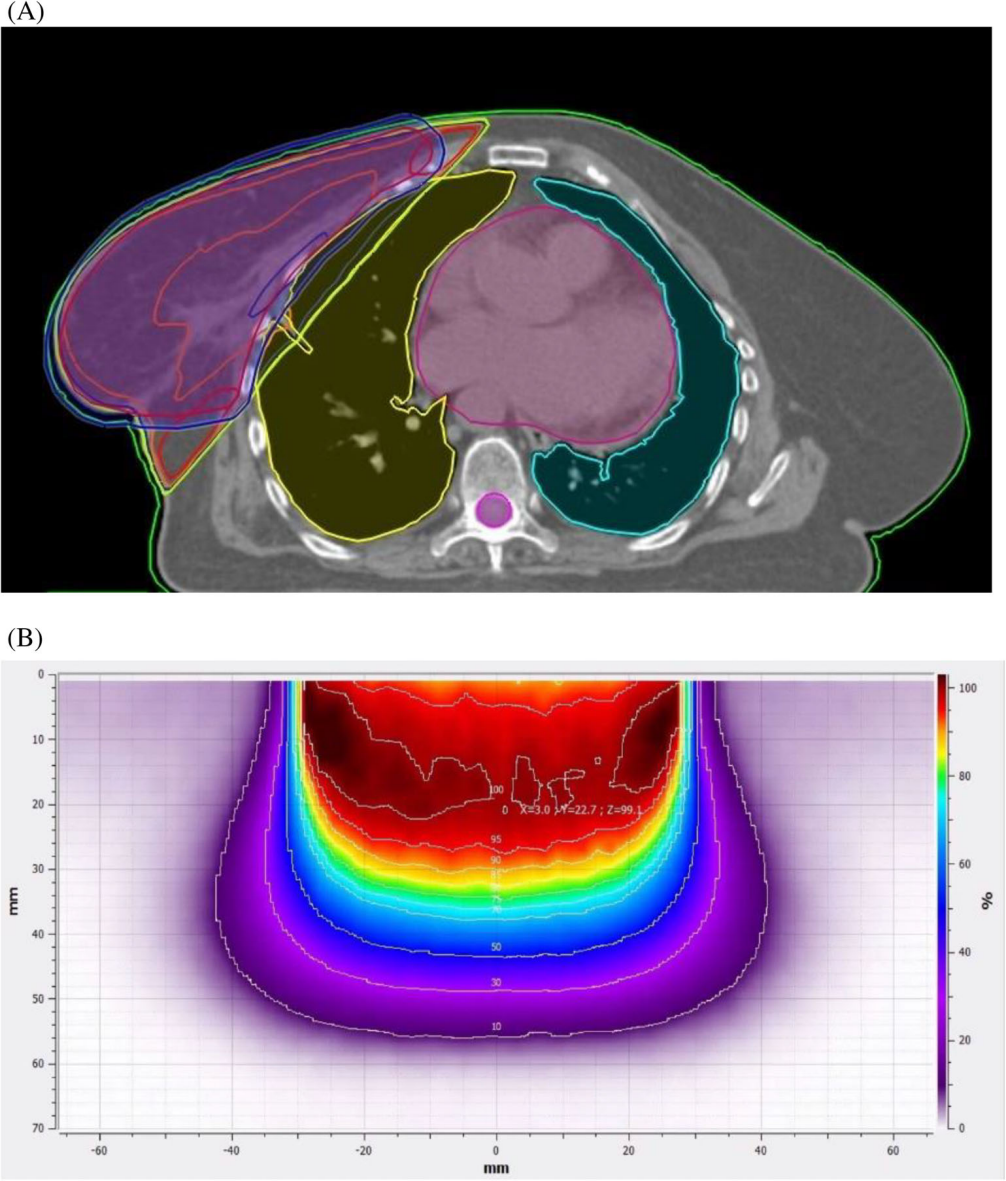
Dose distribution of (A) EBRT, and (B) IORT of a field of 6 cm diameter of 12MEV electron of our device in a representative patient.

Figure 3 compares the dose distribution of EBRT and IORT in a representative patient.

### 3.1 Comparison of the postoperative complications

Comparison of acute and subacute postoperative complications between the IORT and EBRT groups revealed that during the first 24 h after recovery, 60.3% of the IORT group and 51.8% of the EBRT group had low grade and tolerable pain (G1‐G2) (Table 2). More severe pain (G3‐G4) was observed in 9.1% of the IORT group and 3.5% of the EBRT group (*P* = .021). Similarly, less severe pain was observed in the EBRT group one week and three weeks after BCS (*P* < .001). The results of the multivariate analysis revealed that IORT was associated with more severe pain (OR = 1.929, 95% CI: 1.116–3.332), while larger tumor size was associated with milder pain (OR = 0.567, 95% CI: 0.362–0.887). (Table 3).

**TABLE 2:** Characteristics of the included patients.

Variables	EBRT (*n* = 143)	IORT (*n* = 121)	*p* value
Age, years			
Mean (SD)	54.3 (8.9)	55.7 (8.1)	.171
Tumor Size, cm			
Mean (SD)	1.74 (0.64)	1.53 (0.55)	.005
T1, *n* (%)	111 (77.6)	103 (85.1)	.121
T2, *n* (%)	32 (22.4)	18 (14.9)	
Tumor Site, *n* (%)			
Right	69 (48.3)	67 (55.4)	.249
Left	74 (51.7)	54 (44.6)	
Diabetes, *n* (%)	9 (6.3)	14 (11.6)	.130

Abbreviations: EBRT, external beam radiotherapy; IORT, intraoperative radiotherapy; SD, standard deviation.

**TABLE 3:** Acute and subacute complications of the patients who underwent quadrantectomy + SLNB (EBRT group) versus quadrantectomy + SLNB + IORT (IORT group).

Variables	EBRT *n* (%)	IORT *n* (%)	*p* value
Pain after 24 h			
No	64 (44.8%)	37 (30.6%)	
G1‐G2	74 (51.8%)	73 (60.3%)	.021
G3‐G4	5 (3.5%)	11 (9.1%)	
Pain after 1 week			
No	121 (84.6%)	68 (56.2%)	
G1‐G2	22 (15.4%)	51 (42.1%)	<.001
G3‐G4	0	2 (1.7%)	
Pain after 3 weeks			
No	136 (95.1%)	95 (78.5%)	
G1‐G2	7 (4.9%)	26 (21.5%)	<.001
G3‐G4	0	0	
Erythema			
No	115 (80.4%)	94 (77.7%)	
G1‐G2	26 (18.2%)	23 (19%)	.591
G3‐G4	2 (1.4%)	4 (3.3%)	
Edema			
No	124 (86.7%)	102 (84.3%)	
G1‐G2	16 (11.2%)	16 (13.2%)	.868
G3‐G4	3 (2.1%)	3 (2.5%)	
Palpable seroma			
Absent	137 (95.8%)	116 (95.9%)	.979
Present	6 (4.2%)	5 (4.1%)	
Hematoma			
Absent	140 (97.9%)	119 (98.3%)	.578
Present	3 (2.1%)	2 (1.7%)	
Wound infection			
Absent	139 (97.2%)	118 (97.5%)	.592
Present	4 (2.8%)	3 (2.5%)	
Wound dehiscence			
Absent	141 (98.6%)	121 (100%)	.502
Present	2 (1.4%)	0	
Scar retraction			
Absent	141 (98.6%)	119 (98.3%)	.624
Present	2 (1.4%)	2 (1.7%)	
Skin dryness			
No	125 (87.4%)	106 (87.6%)	
G1	14 (9.8%)	13 (10.7%)	.816
G2	4 (2.8%)	2 (1.7%)	
Pruritus			
Absent	121 (84.6%)	100 (82.6%)	.666
Present	22 (15.4%)	21 (17.4%)	

*Note*: *p*‐value <.05 was considered statistically significant.

Abbreviations: EBRT, external beam radiotherapy; G, grade; IORT, intraoperative radiotherapy.

### 3.2 Comparison of the post‐radiation complications

Table 4 shows post‐radiation complications in the IORT and EBRT groups. It is indicated that 93% of the EBRT group experienced low‐grade erythema, compared to 19% of the IORT group (*P* < .001). Skin dryness and pruritus were seen in 30.8% and 26.6% of the EBRT group, and 12.4% and 17.4% of the IORT group, respectively (*P* = .001, *P* = .073). Hyperpigmentation was seen in 48.3% of patients in the EBRT group and 9.9% of the IORT group (*P* < .001). Telangiectasia and breast induration were detected in 1.4% and 14.7% of the participants in EBRT group, while 0.8% and 40.5% of the participants in the IORT group experienced the mentioned complications (*P* = .563 and *P* < .001, respectively). Logistic regression analysis showed that erythema, skin dryness and pruritus, and hyperpigmentation was more severe in the EBRT group, while breast induration was more profound in the IORT group (OR = 4.109, 95% CI: 2.242–7.531) (Tables 5 and 6). Age, tumor site, and diabetes did not affect the severity and rate of postoperative and post‐radiation complications.

**TABLE 4:** Multivariate analysis using binary logistic regression for the association between age, tumor size, tumor site, diabetes, and type of surgery (quadrantectomy + SLNB (EBRT group) vs. quadrantectomy + SLNB + IORT (IORT group)) with postoperative complications.

Variables	Age OR (95% CI)	Tumor size OR (95% CI)	Diabetes OR (95% CI)	Type of Surgery (Ref: QUD+ SLNB) OR (95% CI)
Erythema	1.014 (0.983–1.046)	0.567 (0.362–0.887)	1.086 (0.638–1.849)	0.960 (0.366–2.514)
Acute skin toxicity	1.015 (0.984–1.046)	1.020 (0.656–1.585)	0.757 (0.445–1.286)	1.739 (0.719–4.209)
Skin breaks down	1.033 (0.956–1.117)	0.575 (0.172–1.915)	0.883 (0.214–3.640)	–
Skin necrosis	0.974 (0.940–1.010)	1.057 (0.651–1.716)	0.785 (0.442–1.395)	0.572 (0.184–1.777)
Hyperpigmentation	1.014 (0.983–1.046)	0.567 (0.362–0.887)	1.086 (0.638–1.849)	0.960 (0.366–2.514)
Skin dryness	1.015 (0.984–1.046)	1.020 (0.656–1.585)	0.757 (0.445–1.286)	1.739 (0.719–4.209)
Pruritus	1.033 (0.956–1.117)	0.575 (0.172–1.915)	0.883 (0.214–3.640)	–
Telangiectasia	0.974 (0.940–1.010)	1.057 (0.651–1.716)	0.785 (0.442–1.395)	0.572 (0.184–1.777)
Breast induration	1.014 (0.983–1.046)	0.567 (0.362–0.887)	1.086 (0.638–1.849)	0.960 (0.366–2.514)
Cosmetic outcome	1.015 (0.984–1.046)	1.020 (0.656–1.585)	0.757 (0.445–1.286)	1.739 (0.719–4.209)

*Note*: Pain includes pain after 24 h, one week, and three weeks after the operation. Local complications include erythema, edema, seroma, and hematoma. Wound complications include wound infection and dehiscence.

Abbreviations: CI, confidence interval; OR, odds ratio; Ref, reference; SLNB, sentinel lymph node biopsy; QUD, quadrantectomy.

**TABLE 5:** Acute and subacute complications and outcomes of radiotherapy in the patients who received IORT versus EBRT.

Variables	EBRT *n* (%)	IORT *n* (%)	*p* value
Erythema			
No	6 (4.2%)	95 (78.5%)	
G1‐G2	133 (93%)	23 (19%)	<.001
G3	4 (2.8%)	3 (2.5%)	
Acute skin toxicity			
No	17 (11.9%)	120 (99.2%)	
G1	112 (78.3%)	1 (0.8%)	<.001
G2	14 (9.8%)	0	
G3‐G4	0	0	
Skin breaks down			
No	31 (21.7%)	121 (100%)	
G1	105 (73.4%)	0	<.001
G2	7 (4.9%)	0	
G3‐G4	0	0	
Skin necrosis			
Absent	143 (100%)	121 (100%)	–
Present	0	0	
Hyperpigmentation			
No	74 (51.7%)	109 (90.1%)	
G1	57 (39.9%)	12 (9.9%)	<.001
G2	12 (8.4%)	0	
Skin dryness			
No	99 (69.2%)	106 (87.6%)	
G1	36 (25.2%)	13 (10.7%)	.001
G2	8 (5.6%)	2 (1.7%)	
Pruritus			
Absent	105 (73.4%)	100 (82.6%)	.073
Present	38 (26.6%)	21 (17.4%)	
Telangiectasia			
Absent	141 (98.6%)	120 (99.2%)	.563
Present	2 (1.4%)	1 (0.8%)	
Breast induration			
Absent	121 (84.6%)	72 (59.5%)	<.001
Present	22 (14.7%)	49 (40.5%)	
Cosmetic outcome			
Excellent	16 (11.2%)	36 (29.8%)	
Good	103 (72%)	77 (63.6%)	<.001
Fair	24 (16.8%)	8 (6.6%)	
Poor	0	0	

*Note*: *p*‐value <.05 was considered statistically significant.

Abbreviations: EBRT, external beam radiotherapy; G, grade; IORT, intraoperative radiotherapy.

**TABLE 6:** Multivariate analysis using binary logistic regression for exploring the association of age, tumor size, tumor site, diabetes, and type of radiotherapy (EBRT vs. IORT) with post‐radiation complications.

Variables	Age OR (95% CI)	Tumor size OR (95% CI)	Diabetes OR (95% CI)	Type of radiotherapy (Ref: EBRT) OR (95% CI)
Erythema	1.014 (0.968–1.063)	1.464 (0.738–2.907)	1.251 (0.349–4.482)	0.012 (0.005–0.031)
Acute skin toxicity	1.191 (0.936–1.514)	3.264 (0.224–47.561)	–	–
Skin breaks down	1.014 (0.980–1.048)	0.848 (0.526–1.368)	0.493 (0.142–1.718)	0.114 (0.057–0.230)
Skin necrosis	0.988 (0.959–1.019)	1.068 (0.695–1.641)	0.802 (0.306–2.100)	0.496 (0.291–0.848)
Hyperpigmentation	1.002 (0.969–1.037)	1.429 (0.873–2.339)	0.976 (0.371–2.565)	4.109 (2.242–7.531)
Skin dryness	0.981 (0.945–1.017)	1.120 (0.652–1.922)	1.058 (0.361–3.098)	0.303 (0.156–0.589)
Pruritus	1.014 (0.968–1.063)	1.464 (0.738–2.907)	1.251 (0.349–4.482)	0.012 (0.005–0.031)
Telangiectasia	1.191 (0.936–1.514)	3.264 (0.224–47.561)	–	–
Breast induration	1.014 (0.980–1.048)	0.848 (0.526–1.368)	0.493 (0.142–1.718)	0.114 (0.057–0.230)
Cosmetic outcome	0.988 (0.959–1.019)	1.068 (0.695–1.641)	0.802 (0.306–2.100)	0.496 (0.291–0.848)

*Note*: Skin complications include acute skin toxicity, skin break down, and skin necrosis.

Abbreviations: CI, confidence interval; EBRT, external beam radiotherapy; OR, odds ratio; Ref, reference.

### 3.3 Comparison of the cosmetic outcomes

Excellent, good, and fair cosmetic outcome was seen in 11.2%, 72%, and 16.8% of the patients in EBRT group and 29.8%, 63.6%, and 6.6% of the IORT group, suggesting that cosmetic outcome was significantly better in the IORT group (*P* < .001).

### 3.4 Comparison of outcomes

**FIGURE 4:**
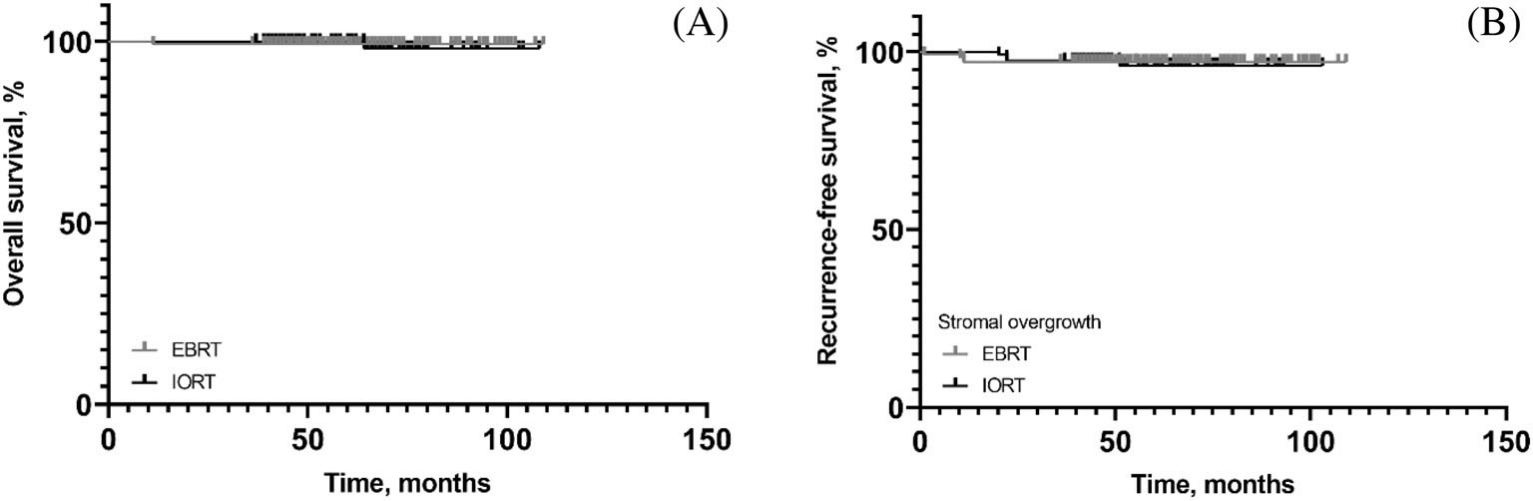
Kaplan–Meier survival plot of Overall survival (A), and recurrence‐free survival (B).

During the study period, patients were monitored for recurrence with a mean follow‐up period of 63.5 (SD 13.49) months. In IORT group, 4 patients (3.3%) experienced recurrence, among which two (1.7%) were local recurrence, one (0.8%) were regional recurrence, one patient (0.8%) had metastasis. Also, 4 patients (2.7%) had recurrence in EBRT group, one (0.7%) were local recurrence, two (1.4%) were regional recurrence and one patient (0.7%) had metastasis. The mean time to recurrence was 19.44 ± 24.3 months in the IORT group and 38.3 ± 18.3 months in the EBRT group, but the difference was not statistically significant (*P* = .136). The mean overall survival and recurrence‐free survival time were 107.15 ± 0.834 (95% CI: 105.52, 108.79) and 98.91 ± 1.623 (95% CI: 95.72, 102.01) months in the IORT group, and 108.32 ± 0.681 (95% CI: 106.98, 109.65) and 105.55 ± 1.51 (95% CI: 102.59, 108.52) months in the EBRT group, respectively. There was no significant difference between the two groups in terms of overall survival (*P* = .953) or recurrence‐free survival (*P* = .56). Kaplan–Meier plots for overall survival and recurrence‐free survival are shown in Figure 4.

## 4 DISCUSSION

This is the first study in Iran comparing both postoperative complications and post‐radiation complications of two different breast cancer radiotherapy methods (IORT vs. EBRT). Our study results indicated that IORT can be considered an acceptable treatment option for early‐stage breast cancers that fulfill the eligibility criteria of our study, accompanying a lower rate of local complications compared to EBRT. Patients receiving IORT experienced more severe pain than those in the EBRT group, one to three weeks after operation. However, we found that erythema, acute skin toxicities, skin break down, hyperpigmentation, skin dryness, and pruritus were more frequent in the EBRT group. The cosmetic outcome was also more desirable in the IORT group. Other postoperative complications such as edema, seroma, hematoma, and wound complications were not significantly different between the IORT and EBRT groups.

We had larger tumors in the EBRT group. But there was no significant difference in tumor size classification based on *T* stage, and this difference did not interfere with treatment techniques and consequently with radiotherapy side effects. First, we compared postoperative complications of patients who received IORT during operation with those in the EBRT group (surgery alone without receiving radiotherapy). Our results showed that IORT was associated with more severe postoperative pain in all the follow ups (24‐h, one week, and three weeks) after the operation. In line with previous studies, palpable seroma was one of the most common postoperative complications in the IORT group, reported in 4.1% of the cases.^30^ The results of our study were consistent with that of Kraus‐Tiefenbacher et al. that found the rate of palpable seroma to be similar between IORT and non‐IORT groups.^36^ However, whether the rate of seroma requiring puncture is different between IORT and non‐IORT groups still remains controversial.^16^, ^29^ The rate of hematoma was 1.7% in our study, which was near to a previous study reporting that 1.4% of IORT patients experienced hematoma.^37^ Wound infection was one of the most common wound complications, occurring in 2.5% of IORT patients. Interestingly, none of these wound infections required intravenous antibiotics. It was in contrary with the TARGIT‐A trial, which indicated that 1.8% of IORT patients needed intravenous antibiotics for wound infection.^16^ This difference may be attributed to the prescription of prophylactic antibiotics for all participates in our study. The results of the multivariate analysis revealed that age, tumor site, and diabetes did not affect postoperative complications. Interestingly, lower tumor size was associated with more severe pain. This can be explained by the fact that there was a predilection to treat patients with lower tumor size with IORT and patients who underwent IORT experienced more severe pain than the EBRT group.

Second, post‐radiation complications of IORT and EBRT were compared. Findings of this survey were consistent with the ELIOT trial, which represented that the IORT group had a lower rate of side effects, including erythema, dryness, hyperpigmentation, pruritus, and pulmonary fibrosis compared to the EBRT group; however, fat necrosis rate was higher in the IORT group.^14^ Furthermore, it has been shown that the Radiation Therapy Oncology Group (RTOG) toxicity grade 3 or 4 was significantly lower in the IORT group.^16^ However, frequency of major toxicity and skin breakdown was similar in the two groups. These minor differences may be attributed to the differences in study designs, including patient characteristics, and also the IORT itself (20 Gy using 50‐kV x‐rays in TARGIT‐A trial^16^ vs. 21 Gy using electrons in ELIOT trial^14^ and our study). Multivariate analysis indicated that none of the demographic variables, including age, tumor size and site, and diabetes affected the rates of post‐radiation complications. Only the method of radiotherapy influenced these complications, indicating that most of the post‐radiation complications were lower in the IORT group.

Our results on the superiority of IORT over EBRT in terms of cosmetic outcomes are in line with previous studies. Keshtgar et al. used BCCT. core software, which is an objective tool to assess the esthetic outcomes following radiotherapy.^38^ They showed that IORT was accompanied with better esthetic results. EBRT patients had lower breast contour during the 5‐year follow up period. EBRT was also associated with more changes in color and scaring of the breast; however, these changes decreased throughout the follow‐up period. Therefore, subjective and objective assessment of cosmetic outcomes following breast radiotherapy showed similar results, favoring the use of IORT.

Our study found no statistically significant difference in recurrence‐free survival and overall survival rates between two groups of patients who received either IORT or EBRT.^39^, ^40^, ^41^ These findings are consistent with a substantial body of previous research, including a meta‐analysis by Wang et al., which also failed to demonstrate a meaningful difference between the two treatment modalities in terms of recurrence‐free survival and overall survival. Our results align with the findings of previous studies, as we also observed a higher rate of local recurrence in the IORT group compared to the EBRT group.^40^, ^42^, ^43^ Contrary to previous studies, our findings revealed a higher incidence of regional recurrence in the IORT group compared to the EBRT group.^14^, ^44^ Several factors may contribute to these variations, such as the sample size of our study and the limited occurrence of recurrence within our study population. Furthermore, our study exclusively focused on patients with luminal A or B breast cancer, setting it apart from previous investigations.

Overall, IORT is accompanied with lower post‐radiation side effects and better cosmetic outcomes; however, the postoperative evaluation did not reveal a significant difference between the IORT and the EBRT groups, except for pain. These findings confirmed that the superiority of IORT over EBRT cannot be attributed to known and unknown confounding factors and can be mainly explained by the difference in radiotherapy methods. This hypothesis was also confirmed by the results of the multivariate analysis, indicating that age, tumor size, and diabetic status of the patients did not increase the risk of most of the post‐radiation complications and only the method of radiotherapy was a determinant of these complications.

Mortality and survival rates of the IORT patients are not different from EBRT patients; however, due to the higher ipsilateral breast tumor recurrence (IBTR) rate in the IORT group, it is necessary to find suitable patients for IORT based on validated guidelines to minimize the risk of IBTR and metastasis.^45^, ^46^, ^47^ We should also consider the effects of these two radiotherapy methods on the quality of life before recommending and choosing an ideal procedure for each patient,^48^ that is, IORT is accompanied with a better quality of life and role functioning.^33^ In addition to its mentioned clinical benefits, IORT is less costly than EBRT from an economics perspective.^49^

Some variables, including field of radiation, history of smoking, allergy, and hypertension may affect the rate of complications after radiotherapy. Nevertheless, history of diabetes, size, and tumor stage are not correlated with radiotherapy complications, which was in line with the results of our study.^50^, ^51^ We tried to minimize the risk of bias by allowing the eligible patients to choose between IORT and EBRT after explaining the benefits and risks. This approach resulted in homogenous distribution of variables, which lower the risk of bias. The major limitation of our study is the possible role of confounders during the allocation treatment groups. For instance, level of education may influence the results of the study. It was shown that patients with a higher level of education are more likely to choose IORT as the treatment option.^52^ A higher educational level may be associated with better skin self‐care, which may affect the results. Therefore, it is necessary to consider the level of education and other influential characteristics of the patients for randomization in future studies. However, the comparison of postoperative complications, showing the insignificant difference between the IORT and the EBRT group, points to the fact that the lower post‐radiation complications of IORT are probably not affected by confounding factors. Also, we set up strict eligibility criteria, yielding to the inclusion of more homogenous patients with very similar demographic and tumor characteristics. Furthermore, data collection and examinations in this study were conducted by trained physicians, which guaranteed the credibility of the findings.

## 5 CONCLUSION

Despite the insignificant difference between postoperative complications of the IORT and the EBRT group, IORT has lower post‐radiation complications and better cosmetic issues in breast cancer patients. Considering these findings and other clinical and economic benefits, IORT might be used as the treatment of choice in a considerable number of patients. However, it is important appropriately choose eligible patients to be treated with this method.

## AUTHOR CONTRIBUTIONS

**Vahid Zangouri:** Investigation (equal). **Amirhossein Roshanshad:** Methodology (equal). **Aliyeh Ranjbar:** Methodology (equal). **Mahsa Izadi:** Methodology (equal). **Sara rajaeifar:** Methodology (equal). **Ali Goodarzi:** Methodology (equal). **Hamid Nasrollahi:** Supervision (equal).

## FUNDING INFORMATION

The project was funded by Vice Chancellor for Research of the Shiraz University of Medical Science [Grant No. 99–01–01‐22758].

## CONFLICT OF INTEREST STATEMENT

The authors declare that they have no competing interests.

## ETHICS STATEMENT

Ethics committee of Shiraz University of Medical Sciences approved this study (ethical code: IR.SUMS.MED.REC.1400.078).

## CONSENT TO PARTICIPATE

The patients consented to participate in this study voluntarily after being explained about our study protocol and objectives. The current study was conducted in accordance with the declaration of Helsinki, and the patients gave their informed and written consent to participate in this study.

## Data Availability

Data is available from the corresponding author upon reasonable request via email.
